# Topics of Public Interest

**Published:** 1914-12

**Authors:** 


### TOPICS OF PUBLIC INTEREST.

**A Relief Fund For Belgian Physicians** is being raised by American Medicine, 18 E. 41st street, N. Y. C. Even the smallest contributions will be accepted. Bear in mind that this comparatively small country, almost entirely free from obstacles such as mountains and large rivers, has been almost literally trampled under foot by a hostile army. This is not a figure of speech. The actual damage to arable land merely from being walked upon by any considerable number of men is enormous. Roadways have been worn out simply from the traffic upon them, not to mention the deliberate destruction of pavements, bridges, etc., as a necessary strategic measure. With the strictest discipline to prevent looting and unnecessary destruction of property, it is inevitable that the country should have been drained of its material resources, and that bombardment, fire and neglect from abandonment should have caused immense loss in buildings and equipment of all kinds. To date, Belgium has suffered more than any other country. Remember what you have read and heard of the damage to our own country, when a tenth of the number of men fought over a territory ten times as large. Try to imagine yourself in the place of the average Belgian physician and do what you can.

**Streets As Play Grounds.** Every driver, even before the days of automobiles has experienced subjective cardiac ectopy on account of the use of the streets as playgrounds. Most cities have been heavily taxed—though still inadequately—to provide substitute playgrounds, but the evil grows. Probably the damage to tires and machinery alone from sudden stops exceeds the amount spent for proper playgrounds, not to mention the inevitable loss of life. Miss Ruth Wilkinson, Organizer of Children's Play for the People's Institute of N. Y., makes a suggestion which is along the lines of similia similibus but which strikes us as eminently sensible till the ideal condition of adequate public playgrounds can be realized. She would close certain streets—not of course main thoroughfares—for certain times each day, to vehicular traffic, putting up signs similar to those used when streets are closed for repairs, and turn these streets into playgrounds. We would add the qualification that, with this provision all children—not omiting those from 21 to 75 years—who use the street from curb to curb for play of any kind should be arrested, taken to the nearest station house and held until their parents or guardians call for them.

---

**The Philadelphia P. G. School of Neurology** announces clinical courses in three sessions of six weeks each, the first beginning December 7. Fee $25, plus amounts necessary to cover additional expenses in serology, pathology, etc. Special short courses of 2-3 weeks may be arranged from June to September. Further information on application to the Dean, Dr. C. K. Mills, 1909 Chestnut Street.

---

**Economic Reaction of the European War on America.** In the September issue, the first published after the outbreak of hostilities, when gloomy predictions were being made generally regarding the effect on this country, we took the optimistic view that there must occur a demand for materials of various kinds that would react favorably on American trade. October 31, over two million pairs of shoes had been ordered, and the New England factories, which represent about 55% of the total production of the U. S., were working a quarter over time as compared with an average of four-tenths capacity. Another order to a Pittsburg firm is for 200,000 pairs for the French delivered by January 1, and 500,000 for the English delivered by April 1, at $3.25 per pair. Horses are being bought by agents of the European powers. A large number of motor trucks have been ordered, including about two millions of dollars to Buffalo alone. Flour, grain and meats have been ordered in large amounts, and even the em-meats have been ordered in large amounts and even the embarrassingly large cotton crop seems to be provided for, partly by direct necessities in Europe, partly as a substitute for linen whose production in Europe is interfered with.

---

**Wood Alcohol Poisoning.** Thirteen deaths among 40 men drinking a concoction of ether, wood alcohol and water, occurred in Bristol, Vt., early in November. The druggist who sold the mixture, Dr. H. D. Bisbee, was threatened with lynching. As we have previously pointed out, deaths or blindness from wood alcohol used commercially, with any reasonable degree of caution, are extremely rare. The great majority of cases of poisoning have occurred either from exposure to fumes without proper ventilation, as in varnishing the interior of beer vats, or from using wood alcohol as an adulterant of beverages. With the publicity given to the danger of wood alcohol, this wholesale destruction of life, due to a man engaged in a profession in which ignorance of the properties of this substance can scarcely be imagined, has but one logical punishment.

---

**Army Medical Corps Examinations** will be held January 11, at points to be selected in accordance with localities from which applications may be received. 20 vacancies exist. Applications should be made promptly to the Adjutant General, U. S. A., as the blanks supplied must be filled in and filed at least 3 weeks prior to the examination. One year's interne service is required as a qualification.

---

**Death of Centenarian.** At the Gettysburg semicentennial we had the pleasure of meeting Micajah Weiss, the oldest veteran, who was born in Dancing Creek, Pa., in 1800. He died September 24, at his home in Beaver Creek, Sullivan Co., N. Y.

---

**Automobile Insurance.** Read your policy carefully and note whether it contains the "deductable" clause by which the company is not only exempt from claims for minor losses but from payment of more than a nominal margin on any loss much short of complete destruction.

---

**Military Gangrene,** long suspected of being an infectious process and thus differing from the conception of non-surgical gangrene, though not, of course, limited to military surgery, is asserted by Drs. James Scarlett and Georges Desjurdins of the American Ambulance Service of Paris, to be due to a specific bacterium, recently discovered. Dr. Henri Weinberg of the Pasteur Institute, is preparing a serum.

---

**The Single Tax and Sanitation.** Surg.-Gen. Wm. E. Gorgas, at a banquet of single-taxers in Cincinnati, spoke on the relation of sanitation and health to poverty. Those interested have asked us to mention the subject and support the Single Tax. The fact that poverty causes disease and death cannot be denied. How far equalization of wealth is practicable or even desirable is a debatable question. Laborers are pretty well agreed that increase of wages has not brought the anticipated relief but equally agreed that the only remedy is to continue raising wages. We approve of a single tax—based on actual income, with such exemptions at a lower limit and such progressive taxation of large incomes as may seem expedient. But we do not think that actual facts warrant the support of what is ordinarily considered the single tax. Real estate is, on the average, not a particularly lucrative business and it requires more capital in proportion to the amount of business done than any other. Why a man making his living from real estate should pay a tax of approximately 25% on his gross earnings and a grocer, dry goods man, or professional worker none, in the direct sense, is not obvious. The present taxation on real estate carries the same confiscatory tendencies as are advocated for the single tax; not to the ideal degree but to the extent that it is cheaper to rent than to own the average home and that the average property holder is usually glad to sell out after a few years at a moderate loss. But, the single-taxer, will say, the idea is to tax only the land itself. There are two replies to be made to this argument: first, the present system of taxation means that if the speculator in vacant land is to come out even, with the same return that he would have from a conservative income investment, he must sell at double the original assessed value within ten years, provided that no improvements have been made. If pavement, sidewalk, water, gas and sewer service, lighting, etc., have been added, he must get about four times the original value. And, the second reply is even more cogent. Land held in common is useful to the state or community only to a certain degree, namely the insurance of an abundance of park and playground room and occupation by public buildings. Waste land, to which anybody can lay claim on payment of ground rent means lack of responsibility, weeds, collections of garbage, ashes and rubbish, criminality, disease, unless the state—or other government—instead of deriving a revenue from it in taxes, undergoes expense to guard and partially improve the land. Absolute title is requisite to the development of property, either for farming or for building in cities and towns. If a man can get a piece of land by paying a ground tax, he may when he feels the need, put up a cheap temporary structure or plow it and plant a crop but he will not, so long as human nature remains what it is, erect a substantial, permanent building or develop the land as it should be for efficient farming.

---

**Oxypathor Conviction.** A federal jury in Vermont has found the vice-president of this company guilty of fraudulent use of the mails. The evidence showed a profit of $500,000 on 45,000

apparatuses, costing $2 each. The active ingredients were coke dust, kaolin, lampblack and iron filings, with traces of antimony, chlorin, sulphur and ammonia. Experts testified that the apparatus would not deliver an electric current, radiant energy or oxygen. Of the 73 diseased conditions enumerated as curable by the apparatus, 23 were functional, mechanic or senile in which mental suggestion is more or less effective and 27 are infectious. On the other hand, patients testified to various wonderful cures, as from diphtheria, pellagra, Bright's disease, etc.

---

**White Slavery.** Out of the mass of hysterical untruth, emerges the address before the International Purity Congress at Kansas City, by Miss Margaret E. Luther, Superintendent of the Florence Crittenden Home of N. Y. Speaking from experience, she states that the majority of girls who go wrong are not alone in the world nor are they necessarily wage earners. Their downfall is due to bad homes and the evil begins early in life. Of 450 girls brought before the N. Y. Women's night court, 289 were not over 18 and 116 were only 16. Stories of locked doors and barred windows—in other words, of literal white slavery—are mostly imaginary; the slavery is psychic. The same factor of lack of good home influences and of early degradation is manifest if the matter is viewed from the standpoint of a male—we can scarcely use the word man. One judge declared that 90% of the sentences for white slavery were imposed on boys less than 22. Contrary to most reformers, Miss Luther realizes that people can not be made good by law. She considers the N. Y. State laws as good and as strong as possible. It is the home influence that needs strengthening. We thing that reform along social lines would be expedited if it were more clearly recognized that, primarily, the male white slaver is what is technically known as a pup. He differs only in degree from the youth who deliberately seeks a wealthy marriage—and note that we do not believe that difference in wealth should interfere with the nátural course of human affections,—from the promotor who specializes on the inexperienced widow and orphan, from the man who plays on his attractiveness to the female sex to secure loans and gifts. Every father should realize that his son has taken his first degree in pimping when he lets some girl pay for his ball tickets.

---

**A Protection Fund** of $30,000 has been appropriated for the protection of girls visiting the San Francisco Exposition. This is a practical philanthropy. The floating population of such large gatherings involves special opportunities and temptations. A large amount of voluntary moral obliquity can not be prevented. We have heard of it even at fraternal and Sunday school conventions. But the deliberate preying upon innocent girls and the dangers due to temporary homelessness and accidental financial difficulties, is largely preventable.

**Bumper Crop for New York.** The press states that the production of this state is unusually high, aggregating for grains, potatoes and apples, a value of over $90,000,000. However, even this production represents a total per capita of only about $9. Persons of mederate means, say $1000-$3000 annual income, usually spend $100-$200 per capita for food alone. Even with the cheapest foods, selected for their physiologic and financial economy, it is almost impossible to reduce the cost of food below ten cents a day—although systematic, collective buying, exclusion of meats, etc., renders it possible to furnish the theoretic caloric equivalents at an expense of not much over five cents a day. But $36.50 per year per capita, is about the minimum cost of food even on a caloric basis, if meat, fruit and butter is included even to a small degree, for persons singly and in families. On this minimum estimate, about $20 a year would have to be spent on the class of products mentioned. If less than this amount were thus expended, instead of reducing the total cost, it would increase it. Now, New York is not a typic agricultural state but it is not in any sense an overcrowded state, even in comparison with other units of the United States and it is far less densely populated, on the average, than most of the European countries. Most of its area is better adapted to some form of agriculture in the broad sense than to anything else, and it is actually so used. Comparatively small areas are devoted to market gardening and while there is considerable stock raising and production of milk and its derivatives, most of the land thus used is also used for crop farming, in alternation. It is something of a shock, in view of the natural tendency to commiserate with European countries that are dependent for their actual food supply upon importations, to realize that, in a wonderful productive year, we have raised only about half of the necessities of life of a vegetable nature.

**Lebanon Hospital Clinics** will be held Wednesday at 3 p. m., by Dr. Parker Syms, Attending Surgeon and Dr. M. R. Bookman, Adjunct Surgeon. The profession is cordially invited. Take West Farms Division of Subway to Jackson Avenue station. See daily bulletin of Academy of Medicine.

**Chlorine Treatment of Buffalo Water.** This method has been in use since September 1. For October, the total bacterial count ranged from 10 to 74 per c. c., none showing colon bacilli, as compared with previous counts averaging ten times as great and with frequent demonstration of colon bacilli. The chemic test shows no odor of chlorine and no complaints have been received since the treatment was begun, though several reports of bad odor and taste from chlorine were received after the public had been informed that such treatment was contemplated but before it was begun. The typhoid incidence is somewhat higher than last year but 70% of the cases are traced to extra-mural sources.

---

**Chlorine in Water.** Since preparing the notice with regard to the chlorine treatment of Buffalo water, popular complaints have become numerous. It is said that the chlorine taste is so strong that it is recognizable even in coffee and that the chemic action of the chlorine destroys clothes washed in it. The best comment on all this is by the Times, to the effect that it is better to have an imaginary taste of chlorine than a real case of typhoid.

---

**Fee for Reporting Tuberculosis.** After January 1, Syracuse physicians will receive one dollar for each case of tuberculosis reported to the local board of health. It should be a matter of common practice to pay a reasonable fee for any similar public duty required of physicians by law and we hope that the principle will be recognized by health officials and legislators in drafting new laws.

---

**Division of Mental Hygiene, U. S. Public Health Service.** A bill has been presented to both houses of congress creating such a division and it is believed that it will pass. We approve of this bill but think that it should be realized that it will involve the Service in quite a distinct line of activity which will require a considerable addition to its personnel, and a corresponding increase of authority and of administrative expense. We have long favored the establishment of this Service as the official health department of the nation, instead of as a branch of the treasury. This opinion is not unanimously held and we feel that this is the time to consider the objections. We hope that they will be overruled but, if not, this bill should not be passed as it would be unwise to extend the Service only to degrade it subsequently by superseding it by a health department of another form.

---

**Back Numbers of Journal Wanted.** The Eighth District Branch of the Dental Society of the State of N. Y. needs, to complete its files, the issues of February 1885 and January-July 1859. It offers various back numbers in exchange. Address A. F. Isham, D. D. S., 85 N. Pearl Street, Buffalo.

---

**Allegany Co.** expects to establish a bacteriologic laboratory.

$2500 has been appropriated by the supervisors for the equipment and first year's salary.

---

**Civil Service Examination.** The U. S. Civil Service Commission, announces an examination December 15, for epidemiologist (male) at a salary of $4000. A collegiate and a medical degree, and at least five years' experience, including field studies and laboratory technic, as well as five years' official service under Federal, state or municipal authorities, are required. The rather unnecessary statement is made that applicants must not be less than 25 years old. Neither may they be over 40. Applicants should send to the Commission at Washington or to the Custom House at New York, for blanks No. 304 and 2095.

---

**Buffalo Branch of National Relief Fund.** Wm. H. J. Cole, British Vice Consul at Buffalo, asks us to publish a notice of this branch. Funds will be devoted to the relief of the Belgian refugees and to the alleviation of military and civil distress. In anticipation of such criticisms as have appeared in the news papers, through misapprehension, we wish it understood that funds contributed will be used in such a way as to involve no violation of either the letter or the spirit of neutrality.

---

**American Ambulance Hospital.** Over $6000 have been collected by the Buffalo Branch.

---

**University of Buffalo Alumni,** to the number of 75, representing all departments, met in Jamestown, November 18, for a banquet, at which Dr. George E. Ellis of Dunkirk acted as toastmaster. Drs. James A. Gibson, Willis G. Gregory, Daniel H. Squire, George F. Cott, D. H. McCoy and Lesser Kauffmann made addresses.

---

**Foot and Mouth Disease.** We wish merely to refer to the value of the daily press service in noting the progress of the epidemic. It is impossible to treat such a subject adequately in either a monthly or a weekly medical journal, except in review and this we understand will be published in the State Health Bulletin.

**Participation of Canadian Universities in the War.** Our associate editor, Dr. J. George Adami, communicates the following:

McGill University with 1500 students has already more than 900 enrolled in the University battalion, of which more than 700 are students, the rest professors and graduates. Toronto is equally keen. Queen's University out of her graduate and under graduate students has already supplied an Engineering

Corps which went with the first contingent and is now at Salisbury Plain. The Medical Faculty of McGill University has offered the British War Office the personnel of a general hospital of 520 beds, and we heard only last week from London that the proposal is very favorably entertained. Further at Toronto and McGill, and other Universities, the medical schools are offering to the two final years that service in the Army Medical Corps will count in place of clinical medicine and surgery in the hospitals, while for the fifth year they are arranging that those who volunteer for the front shall have special courses in Obstetrics and the specialties with examintion in the same before their departure, so that at the end of the session they may receive their degrees upon application.

